# Black Cumin Essential Oil as an Active Stabilization Component of Rapeseed Oil During Deep-Fat Model Heating

**DOI:** 10.3390/foods14132238

**Published:** 2025-06-25

**Authors:** Dominik Kmiecik, Aleksander Siger, Katarzyna Kuraszyk

**Affiliations:** 1 Department of Food Technology of Plant Origin, Poznań University of Life Sciences, 31 Wojska Polskiego St., 60-624 Poznan, Poland; katarzynakuraszyk@gmail.com; 2 Department of Food Biochemistry and Analysis, Poznań University of Life Sciences, 31 Wojska Polskiego St., 60-634 Poznan, Poland; aleksander.siger@up.poznan.pl

**Keywords:** black cumin essential oils, antioxidant effect, heating oils, oils thermal stability, triacylglycerol polymerization, rapeseed oil

## Abstract

The aim of this study was to evaluate the potential of black cumin essential oils to reduce the degradation of rapeseed oil during heating. Rapeseed oil was heated without addition and with the addition of black cumin essential oil (200 ppm, 500 ppm, and 1000 ppm), and with synthetic antioxidant TBHQ (200 ppm). The heating was carried out at 170 °C ± 10 °C for 6 h, in a deep-fat heating model. In all samples, changes in fatty acid profile, lipid-nutritional quality indices (PUFA/SFA ratio, atherogenicity index, thrombogenicity index, and hypocholesterolemic/hypercholesterolemic ratio), tocopherol and phytosterol content, total polar compound content, and triacylglycerol polymers were determined. The heating process led to oil degradation, which depended on the amount and type of additive used. The greatest changes were observed in the control sample (without additives). The addition of TBHQ or 200 ppm of black cumin essential oil reduced the adverse transformations to a similar level. Higher additions of black cumin essential oil led to a significant improvement in the quality of heated oils. The best results were obtained with the addition of 1000 ppm of black cumin essential oil.

## 1. Introduction

Rapeseed oil is one of the most widely produced and consumed edible oils in the world. Its popularity stems from its favorable fatty acid profile (including oleic, linoleic, and linolenic acids), a balanced n6/n3 fatty acid ratio (2:1), and its phytosterol content. Monounsaturated fatty acids (MUFA), mainly oleic acid, account for 63–75%, while polyunsaturated fatty acids (PUFA), primarily linoleic and linolenic acids, constitute 20–29%. The high content and optimal ratio of unsaturated fatty acids are considered highly desirable from a nutritional perspective. However, the high PUFA content also makes rapeseed oil more prone to oxidation, especially during high-temperature processing [1].

One of the most common technological applications of vegetable oils is their use in the frying process. During thermal processing, oil is the medium that transfers heat from the heating element to the heated product. Due to the favorable thermal properties of fats, the product is heated evenly and does not burn. Frying is one of the most commonly used techniques for the thermal treatment of food, both in the home and in foodservice establishments. It is also popular in the food industry, as a method of producing convenience foods or preserving food [2]. One of its main advantages is the product’s unique sensory characteristics, which are highly appreciated by consumers. Fried products have a characteristic golden color, taste, aroma, and texture, which is very difficult to obtain in other cooking methods [3]. In addition, the frying process is fast, economical, and can be applied anywhere. A wide variety of foodstuffs can be fried, both of animal and vegetable origin, such as meats, vegetables, cheese, fruit, cakes, and more. The frying process can be carried out at different temperatures, most often between 170 °C and 230 °C, and in different ways. Frying can be classified according to the amount of oil used into frying with a small amount of oil (pan frying), a medium amount (shallow frying), and a large volume of oil (deep frying). Despite the differences in the method or type of oil used, oil degradation is observed during frying.

Degradation of oil during frying occurs mainly through oxidation, hydrolysis, and thermal processes (primarily polymerization and cyclization) [4]. One of the first reactions observed during thermal processes in oil is oxidation. The oxidation reaction results in the formation of compounds of different molecular weights in the oil. Volatile compounds, aldehydes, ketones or hydrocarbons, are classified as low-molecular-weight molecules than triacylglycerols. Polymerization products (dimers, trimers, and oligomers) are high-molecular-weight compounds, typically resulting from condensation reactions of oxidation reaction products [5,6]. The degradation processes involve not only fatty acids but also other compounds present in the oils, which are included in the unsaponifiable fraction. These compounds include tocopherols, phytosterols, and phenolic compounds [7,8].

As both low- and high-molecular-weight compounds are formed as a result of oxidation, one possibility for counteracting oil degradation during heating is the use of antioxidants. Antioxidants are compounds that, through various mechanisms, prevent oxidation reactions (metal helation, reactive oxygen species scavenging) or terminate radical chain reactions by neutralizing free radicals. Antioxidants found in oils include tocochromanols (tocopherols or tocotrienols), phenolic compounds, as well as carotenoids, squalene, or sesamin. Antioxidants are often added to prolong the oil quality and prevent oil degradation during frying [6]. Synthetic antioxidants such as BHA (butylhydroxyanisole), BHT (butylhydroxytoluene), TBHQ (tert-butylhydroquinone), gallates, or tocopherols are most commonly used. In many countries, rosemary extracts can also be used as an additive [9]. However, the use of synthetic antioxidants raises concerns among consumers and is often ineffective during prolonged frying. Therefore, many researchers are investigating the potential of phenolic compounds obtained from plant extracts for this purpose [10,11]. Despite their suitable activity, the possibility of dissolving them in oils becomes a problem, as well as technical problems in frying. Their addition may also affect the sensory properties of fried products and complicate fryer maintenance. An optimal solution would involve additives with antioxidant properties that are highly soluble in fats. Examples of such additives include antioxidant-rich oils or essential oils containing antioxidant or antiproliferative substances.

Black cumin seeds and black cumin oil have a favorable fatty acid profile (containing 80–90% of unsaturated fatty acid). The predominant PUFA is linoleic acid (50–70%), while oleic acid is the main MUFA (15–20%). Black cumin oil is also rich in bioactive compounds, including sterols and antioxidants [12,13,14]. The main antioxidant compounds include tocopherols and phenolic compounds, particularly thymoquinone and carvacrol [15]. The composition and antioxidant properties of black cumin oil have been extensively described in the literature. Black cumin oil can extend food shelf life and inhibit the growth of pathogenic microorganisms [16,17,18], enhance the sensory properties of food [19,20], and protect oils against oxidation [17,21,22].

Black cumin essential oil is a mixture of biologically active compounds, valued for its complex composition of volatile constituents and strong antioxidant properties. Thymoquinone, carvacrol, and p-cymene are terpenes found in black cumin essential oils [21,23]. These compounds contribute to both the antioxidant activity of the essential oil and its unique sensory qualities. Among natural additives, essential oils are particularly attractive due to their lipophilic nature and good solubility in edible oils, which allows them to overcome the solubility limitations often observed with plant extracts [24].

Due to its high polyunsaturated fatty acid (PUFA) content, the addition of black cumin oil to frying oils increases PUFA levels, potentially leading to faster degradation of the frying medium. The use of essential oil instead of whole oil can eliminate this issue. Moreover, black cumin essential oil contains antioxidants, exhibits proven antioxidant activity, and can be used as an additive to counteract degradation during frying. Its non-polar nature facilitates dosing and mixing with oils for frying. This may support its future application and mitigate the higher cost of oils with natural additives such as essential oils. While the chemical composition and antioxidant properties of black cumin essential oil are well documented, there is a notable lack of studies evaluating its effectiveness in thermal stabilization. Current scientific research primarily focuses on the effects of various extraction methods on the properties of black cumin oil and its essential oil. Although the composition and bioactive compounds of the oil are well characterized, investigations into its application and efficacy under high-temperature processing conditions remain limited. Therefore, the aim of this study was to evaluate the potential of black cumin essential oils as an effective additive for the thermal stabilization of rapeseed oil and to compare performance with that of the synthetic antioxidant TBHQ, which is commonly used in frying oils. Due to increasing consumer demand for natural solutions and concerns over the safety of synthetic antioxidants, essential oils have emerged as promising alternatives for improving the oxidative stability of oils.

## 2. Materials and Methods

### 2.1. Materials

Refined rapeseed oil was purchased from a retail chain in a 3 L volume bottle (Bunge S.A., Kruszwica, Poland). The essential oil was extracted using a hydrodistillation method from unrefined oil from black cumin seeds (*Nigella sativa* L.), supplied directly from the manufacturer SemCo., Śmiłowo, Poland. The synthetic antioxidant tert-butylhydroquinone (TBHQ) was purchased from Merck Life Science, and its purity was 97%. The chemical reagents used in the analyses had ACS, HPLC, or GC grade.

### 2.2. Hydrodistillation and Composition Analysis of Black Cumin Essential Oil

Black cumin essential oil was extracted from cold-pressed black cumin oil following the procedure described by Edris [25]. To a flask containing 250 mL of cold-pressed black cumin oil, 250 mL of distilled water was added, and the mixture was hydrodistilled for four hours using a Clevenger-type apparatus. 1 mL of essential oil per 1 L of cold-pressed oil was obtained. The obtained oil was sealed under a nitrogen atmosphere and stored at 4 °C for 5 days until the preparation of mixtures with rapeseed oil. The composition of black cumin essential oil was analyzed by the GC-MS technique according to the previously described method [26]. Essential oil components were characterized by comparing their mass spectral profiles with entries in the NIST05 database. Compound identification in the ChromaTOF Version 4.60.8.0 software was set at a match factor threshold of 800, corresponding to an 80% similarity in mass spectra. The retention indices (RI) were calculated using a homologous series of n-alkanes (C9–C14) as reference standards.

### 2.3. Oil Sample Preparation and Thermal Treatment

Five 1 L rapeseed oil samples were prepared for the study under identical conditions and at the same time. The first sample was rapeseed oil without an additive (control sample). The next four samples were oil with the addition of synthetic antioxidant TBHQ (200 ppm) and black cumin essential oil (200 ppm, 500 ppm, and 1000 ppm). Concentrations were chosen based on previous studies [27] and the sensory acceptability of the resulting blends. Higher additives significantly affected the sensory characteristics of the blends. The lowest concentration (200 ppm) was chosen for comparison with the maximum allowable additive dose for TBHQ. Additives were added directly to the rapeseed oil, manually mixed, and shaken for 10 h at 180 rpm with an amplitude of 10 mm using a rotary shaker (Steinberg Systems SBS-LAB-125, Steinberg Systemsexpondo Gmb, Berlin, Germany). The samples were shaken to distribute the additive evenly throughout the volume of oil. The samples were stored in a refrigerator at 5 °C, for 2 days, out of light, in dark bottles until analysis.

The heating process was carried out at 170 °C ± 10 °C for 6 h, in a deep-fat heating model. During heating, the oil was in static mode. Oil samples were heated in a volume of 200 mL, in 250 mL glass beakers with a diameter of 7 cm, wrapped in aluminum foil to maintain a constant temperature. The surface-to-volume ratio of the oil was 0.01924 m^2^/L. During the process, the temperature was controlled by the device and by independent external thermometers. Each sample was heated in two independent repetitions on a CAT H 30/30 D heating plate. After the heating process, the samples were cooled, sealed in airtight containers with a nitrogen atmosphere, and stored in a freezer, out of light, at −28 °C until further analysis. All analyses were conducted within 14 days after sample collection.

### 2.4. Characterization of Oil Samples

Characterization of unheated oils included determination of the peroxide value (meq02/kg oil) [28], anisidine value [29], and acid value (mg KOH/g oil) [30].

### 2.5. Fatty Acid Composition Analysis

The fatty acid composition was determined following the AOCS Official Method Ce 1h-05 [31]. An oil sample (50 mg) was dissolved in hexane and transesterified with sodium methylate to obtain fatty acid methyl esters (FAME). The resulting FAME were analyzed using a gas chromatograph, Agilent 7820A GC, equipped with a flame ionization detector (FID) (Agilent Technologies, Santa Clara, CA, USA) and an SLB-IL111 capillary columns (Supelco, Bellefonte, PA, USA) (100 m, 0.25 mm, 0.20 mm). FAME separation was performed under the following conditions: oven temperature initially set at 150 °C, increased to 200 °C at a rate of 1.5 °C/min; the injector and detector temperatures set at 250 °C; split ratio 1:10; helium was used as the carrier gas at a flow of 1 mL/min. The FAME were identified by comparing retention times with those of a commercial FAME standard mixture (grain fatty acid methyl ester mix, Supelco, Bellefonte, PA, USA). The results were expressed as a percentage of total fatty acids.

### 2.6. Calculated Iodine Value—CIV

The iodine value (CIV) of the oil samples was estimated in accordance with the AOCS Official Method Cd 1c-85 [32] and was calculated directly from the determined fatty acid composition. The calculation is based on the proportions of unsaturated fatty acids, specifically hexadecenoic, octadecenoic, octadecadienoic, octadecatrienoic acid, as well as eicosenoic and docosenoic acid. The CIV was calculated using the standard formula:

(1) CIV = (% hexadecenoic acid × 0.950) + (% octadecenoic acid × 0.860) + (% octadecadienoic acid × 0.1.732) + (% octadecatrienoic acid × 2.616) + (% eicosenoic acid × 0.785) + (% docosenoic acid × 0.723)

### 2.7. Nutritional Lipid Quality Indices

Using the fatty acid profile, the indices of lipid nutritional quality of the oils were determined. The fatty acid profile was calculated: Polyunsaturated Fatty Acid/Saturated Fatty Acid index (PUFA/SFA), Index of Atherogenicity (IA), Index of Thrombogenicity (IT), and Hypocholesterolemic/Hypercholesterolemic (HH) Ratio [33].

The IA index was calculated using the standard formula:

(2) IA = [C 12:0 + (4 × C 14:0) + C 16:0]/(ΣMUFA + Σn-6 PUFA + Σn-3 PUFA)

The IT index was calculated using the standard formula:

(3) IT = (C 14:0 + C 16:0 + C 18:0)/[(0.5 × ΣMUFA) + (0.5 × Σn-6 PUFA) + (3 × Σn-3 PUFA) + (Σn-3 PUFA/Σn-6 PUFA)]

The HH ratio was calculated using the standard formula:

(4) HH = (cis − C 18:1 + ΣPUFA)/(C 12:0 + C 14:0 + C 16:0)

### 2.8. Tocopherols and Plastochromanol-8 (PS-8) Analysis

The tocopherols and plastochromanol-8 (PC-8) content were determined as previously described [34]. For analysis, oil samples were dissolved in n-hexane and injected into a Waters HPLC system (Waters, Milford, MA, USA) equipped with a fluorimetric detector (Waters 474) and a photodiode array detector (Waters 2998 PDA). Separation of tocopherols and plastochromanol-8 was carried out using a LiChrosorb Si 60 column (250 × 4.6 mm, 5 μm, Merck, Darmstadt, Germany), thermostatted at 20 °C using a Jetstream 2 Plus column oven. The mobile phase consisted of a mixture of n-hexane and 1,4-dioxane (96:4, *v*/*v*), with a flow rate of 1.0 mL/min. The injection volume was 10 μL. The excitation wavelength was set at λ = 295 nm, and the emission wavelength at λ = 330 nm. Quantitative analysis was performed using the 2475 fluorimetric detector (Waters, Milford, MA, USA), while the photodiode array detector served as an additional tool for the identification of tocochromanols based on UV-Vis spectra. The analyses were performed in duplicate, and the results were expressed in mg/100 g of oil. Tocochromanol standards with a purity above 95% were purchased from Calbiochem–Merck4Biosciences (Darmstadt, Germany). The standard of plastochromanol-8 (PC-8) was prepared in-house by isolating it from flaxseed oil according to the methodology described in earlier studies [35].

### 2.9. Phytosterols Analysis

Phytosterols content and composition were analyzed according to the procedure described by Raczyk et al. [36]. Fifty mg of oil samples, along with 50 µL of internal standard (commercially available standards 5α-cholestane, c = 50.2 mg/25 mL, Sigma-Aldrich, St. Louis, MO, USA), were saponified with 1 M KOH in methanol. Saponification was carried out at room temperature for 18 h. The unsaponifiable fraction was extracted in triplicate using a hexane/MTBE mixture (10 mL, 1:1, *v*/*v*) followed by solvent evaporation under a nitrogen stream, which prevents the oxidation of the isolated sterols. Subsequently, sterols were derivatized by silylation with BSTFA containing 1% TMCS (0.4 mL) and pyridine (0.1 mL) (Sigma-Aldrich, St. Louis, MO, USA) for 24 h at 5 °C. Sterol analysis was performed using an Agilent 7820A GC (Agilent Technologies, Wilmington, DE, USA) equipped with a flame ionization detector (FID) and a DB-35MS capillary column (Agilent Technologies) (30 m, 0.20 mm, and 0.33 µm). Separation of phytosterols was achieved under the followed conditions: injector and detector temperatures were set at 250 °C; helium served as the carrier gas at a flow rate of 1 mL/min, split ratio 1:100. The oven temperature program was initiated at 100 °C, held 5 min, then increased to 250 °C at a rate of 25 °C/min, and finally ramped to 290 °C at 3 °C/min. Phytosterols were identified by comparing their retention times (relative to the internal standard 5a-cholestane) with those of commercially available standards: brassicasterol, campesterol, campestanol, β-sitosterol, and avenasterol (Larodan, Solna, Sweden). The results were expressed in mg/g of oil.

### 2.10. Total Polar Compounds (TPC) Analysis

The total polar compounds in the oil samples were determined following the AOCS Official Method 982.27 [37]. The oil samples (2.5 g) were dissolved in toluene (20 mL) and applied to a glass column packed with silica gel (Sigma-Aldrich, silica gel 60, 63–200 µm). A nonpolar fraction was eluted with a mixture of hexane and diisopropyl ether (82:18 *v*/*v*) and was collected. The solvent was evaporated under reduced pressure at 40 °C using a rotary evaporator (Rotavapor R-210, BÜCHI Labortechnik AG, Flawil, Schweiz), and nonpolar fraction was weighted. The polar components content, as % (weight/volume) was calculated by the formula:

(5) Polar components, % = [(E − A)/E] × 100

where A = g nonpolar fraction; E = g sample in 20 mL aliquot (ca 1 g).

### 2.11. Polymerized Triacylglycerols (PTAG) Analysis

The composition of triacylglycerols was determined in the polar fraction of the oil. Separation of the oil into a polar and non-polar fraction was carried out as described previously [38]. The polar fraction was analyzed using a HPSEC methodology and an Infinity 1290 HPLC (Agilent Technologies, Santa Clara, CA, USA) equipped with ELSD (Evaporative Light Scattering Detector) and two connected Phenogel columns (100 Å and 500 Å, 5 µm, 300 × 7.8 mm, Phenomenex, Torrance, CA, USA), connected in series. Separation of the polymer was carried out under the following conditions: detector temperature 30 °C, detector pressure 2.5 bars, column compartment temperature 30 °C, injection sample volume 1 mL. The analysis was performed using an isocratic method with dichloromethane (DCM) as the mobile phase at a flow rate of 1 mL/min. The total analysis time was 30 min. The results were expressed in mg/g of oil.

### 2.12. Statistical Analysis

All results are expressed as mean values, calculated from two replicates for unheated samples and four replicates for heated oils. Mean values and standard deviations were calculated with Microsoft Office Excel 2019 (Microsoft Corporation, Redmond, WA, USA). Statistical analysis, including the calculation of standard errors and determination of significant differences between means (*p* < 0.05) using one-way ANOVA followed by Tukey’s post hoc test, was performed with STATISTICA PL 13.3 (Dell Software Inc., Round Rock, TX, USA). Principal component analysis (PCA) was conducted using the open-source R software (v4.1), employing the FactoMineR (v2.4) and factoextra (v1.0.7) packages.

## 3. Results and Discussion

### 3.1. Characterization of Black Cumin Essential Oil

GC-MS analysis confirmed the presence of 15 compounds (Table 1). The main components of the essential oil extracted during hydrodistillation were p-cymene, α-thujene, thymoquinone, and trans-4-methoxythujane, which together accounted for 73.03% of all identified compounds. The results obtained are consistent with those reported in earlier studies [27]. However, despite similar levels of p-cymene and α-thujene, the proportion of thymoquinone was 0.4 times lower. Variability in the composition of black cumin essential oil is a well-known phenomenon, and the proportion of individual compounds can fluctuate widely. The chemical composition of the oil is influenced by multiple factors, including the cultivation environment, geographic origin, extraction technique, and raw material source.

### 3.2. Characterization of Oils

The refined rapeseed oil was of high quality. The low peroxide value (1.03 meq 02/kg oil), anisidine value (0.01), and acid value (0.06 mg KOH/g oil) complied with the requirements for refined oils defined by the Codex Alimentarius [39]. The addition of antioxidants did not cause statistically significant changes in the oxidation and hydrolysis parameters of the oils, with the exception of the sample containing 1000 ppm of black cumin essential oil (BC1000). In this sample, the peroxide value increased to 1.27 meq02/kg, which is attributed to the addition of the essential oil. Both black cumin seeds, oils, and essential oils are known to have relatively high levels of peroxide value; thus, their addition directly affects the peroxide value of the prepared mixtures [40]. Changes in the main indices of fresh rapeseed oils with and without antioxidant addition are presented in Table 2.

### 3.3. Fatty Acid Composition Analysis

**Figure 1:**
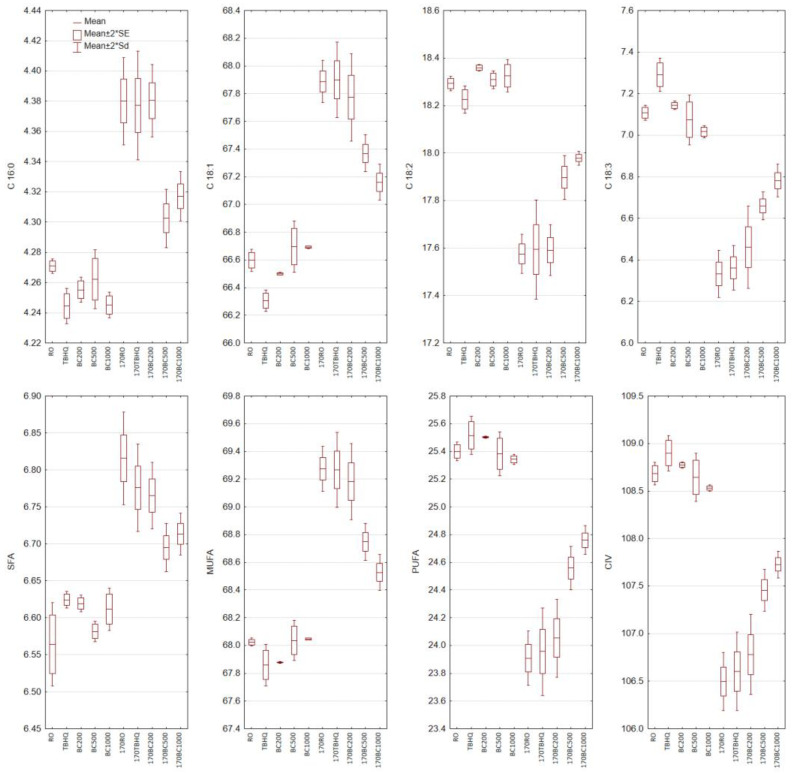
Changes of fatty acid composition (%) in non-heated and heated rapeseed oil with the addition of black cumin essential oil. SFA—saturated fatty acid, MUFA—monounsaturated fatty acid, PUFA—polyunsaturated fatty acid, CIV—calculated iodine value. RO—rapeseed oil without addition, TBHQ—rapeseed oil with addition 200 ppm of TBHQ, BC200—rapeseed oil with addition 200 ppm of black cumin essential oil, BC500—rapeseed oil with addition 500 ppm of black cumin essential oil, BC1000—rapeseed oil with addition 1000 ppm of black cumin essential oil, 170RO, 170TBHQ, 170BC200, 170BC500, 170BC1000—rapeseed oils with addition of TBHQ and black cumin essential oil after 6 h of heating at 170 °C. * SE—standard error, Sd—standard deviation.

The fatty acid composition of the prepared oils was characteristic of rapeseed oil. More than 93% of the fatty acids were unsaturated. The highest share was characterized by oleic acid (C 18:1) (66.6%). Linoleic acid (C 18:2) and linolenic acid (C 18:3) accounted for 18.29 and 7.11%, respectively. The main saturated fatty acids (SFA) were palmitic acid (C 16:0) at 4.27% and stearic acid (C 18:0) at 1.57% (Figure 1). The addition of TBHQ and black cumin essential oil did not alter the fatty acid profile. Changes in the fatty acid profile were observed after the heating process in all samples. However, these changes depended on the type and concentration of the additive used. The greatest changes were observed in rapeseed oil heated without additives, as well as in samples with TBHQ and 200 ppm of black cumin essential oil. In these samples, the share of palmitic, oleic, linoleic, and linolenic acids was similar after heating. The most significant loss was observed in the share of linolenic acid, followed by linoleic acid. However, the addition of 200 ppm of black cumin essential oil inhibited the loss of linolenic acid to a greater extent than TBHQ. In oil samples with 500 and 1000 ppm of black cumin essential oil, higher stability of polyunsaturated fatty acids was observed compared to other samples. The loss in share of linoleic acid was 1.8 to 2 times lower for oils with the addition of 500 and 1000 ppm of black cumin essential oil, respectively. For linoleic acid, the losses were 1.8 to 3 times lower for the corresponding unheated samples in oil with the addition of 500 and 1000 ppm of black cumin essential oil, respectively.

For saturated fatty acids, mainly palmitic acid, and monounsaturated fatty acid, mainly oleic acid, an increase in their share was observed in all heated samples, compensating for the decrease in the share of polyunsaturated acids. As before, the greatest differences were observed in the samples without additives and those with TBHQ and 200 ppm black cumin essential oil.

An increase in SFA and oleic acid is a common phenomenon resulting from the assay method used. However, oxidation is one of the first reactions affecting fatty acids during heating. The rapid increase in temperature, supplies of energy necessary for the initiation and progression of oxidation, and the greater susceptibility of polyunsaturated fatty acids to oxidation lead to their degradation. The rate of oxidation depends on the degree of unsaturation and increases with the number of double bonds in the fatty acid chain [41].

### 3.4. Nutritional Lipid Quality Indices

As the majority of vegetable oils are the main source of unsaturated fatty acids in our diet, their quality is an important factor during the extraction process and in culinary use. In addition to their content of suitable fatty acids, the nutritional value of fats can be presented using indices of lipid nutritional quality. These indices determine the ratio between saturated and unsaturated fatty acids and allow evaluation of their pro-health or pro-inflammatory effects [33,42]. In all lipid indexes, unsaturated fatty acids play the most important role. They are defined as an antiatherogenic factor, in contrast to saturated fatty acids (lauric, myristic, and palmitic), which are calcified as proatherogenic. Therefore, the degradation of polyunsaturated fatty acids significantly contributes to the deterioration of these indices [33]. One of the simplest indicators of the nutritional value of fat is the PUFA/SFA ratio. This ratio assumes that all PUFAs contribute to lowering of LDL cholesterol, while all SFAs increase it. The higher the PUFA/SFA ratio, the higher the nutritional quality of fat. More specific differentiators are the Index of Atherogenicity (IA) and the Index of Thrombogenicity (IT) proposed by Ulbritcht and Southgate [43]. IA is more closely related to the likelihood of atherosclerosis, while IT shows a tendency to form clots in blood vessels. The relationship between hypocholesterolemic and hypercholesterolemic fatty acids is also represented by the HH index.

**Figure 2:**
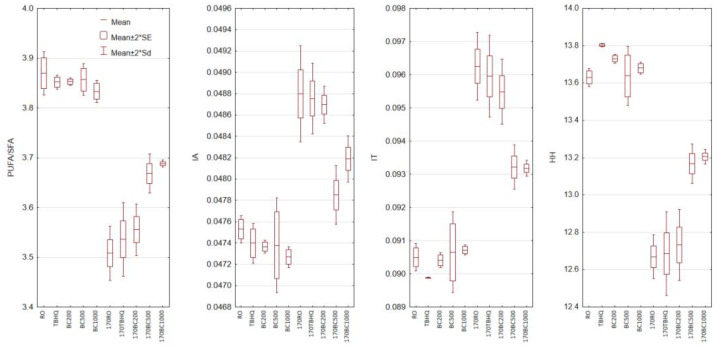
Changes in the indices of lipid nutritional quality of non-heated and heated rapeseed oil with the addition of black cumin essential oil. PUFA/SFA—polyunsaturated and saturated fatty acid ratio, IA—index of atherogenicity, IT—index of thrombogenicity, HH—hypocholesterolemic/hypercholesterolemic ratio. RO—rapeseed oil without addition, TBHQ—rapeseed oil with addition 200 ppm of TBHQ, BC200—rapeseed oil with addition 200 ppm of black cumin essential oil, BC500—rapeseed oil with addition 500 ppm of black cumin essential oil, BC1000—rapeseed oil with addition 1000 ppm of black cumin essential oil, 170RO, 170TBHQ, 170BC200, 170BC500, 170BC1000—rapeseed oils with addition of TBHQ and black cumin essential oil after 6 h of heating at 170 °C.

In both unheated and heated samples, the following indices were determined based on fatty acid composition: PUFA/SFA ratio, IA, IT, and HH ratio (Figure 2). Unheated rapeseed oil had a high PUFA/SFA ratio (3.86) and HH ratio (13.72), along with low IA (0.05) and IT (0.09). Heating the oils led to a deterioration in all analyzed samples. For PUFA/SFA and HH ratio a decrease was observed, while an increase was observed for IA and IT. The greatest changes in indexes were observed in rapeseed oil without an antioxidant and with the addition of TBHQ. The addition of 200 ppm black cumin essential oil resulted in similarly high changes for the IA and HH ratios, and lower changes for the PUFA/SFA ratio and IT. This could indicate that even a small addition of black cumin essential oil, which counteracts fatty acid degradation, may help maintain the oil’s ability to prevent clot formation in blood vessels. At the same time, it does not protect against a decrease in anti-atherosclerotic properties.

The addition of 500 and 1000 ppm of black cumin essential oil reduced the negative effects of the thermal process on the value of all analyzed indices. Although a deterioration in the quality of the nutritional oils was observed, it was between 1.5 and 1.8 times lower than that of the other heated oil samples. The addition of 1000 ppm black cumin essential oil was more effective in minimizing the decrease in PUFA/SFA ratio. While the addition of 500 ppm better inhibited the increase in IA. For the IT and HH ratio, the addition of both 500 and 1000 ppm resulted in a similar quality of the heated oils.

### 3.5. Tocopherols Content

Tocopherols are the most common antioxidants present in vegetable oils. Their content and composition largely determine the stability of oils during storage and thermal processing. The main tocopherols present in rapeseed oil are γ- and α-tocopherol. β- and δ-tocopherol, as well as plastochromanol-8 (PC-8), are also present in smaller amounts [34].

**Figure 3:**
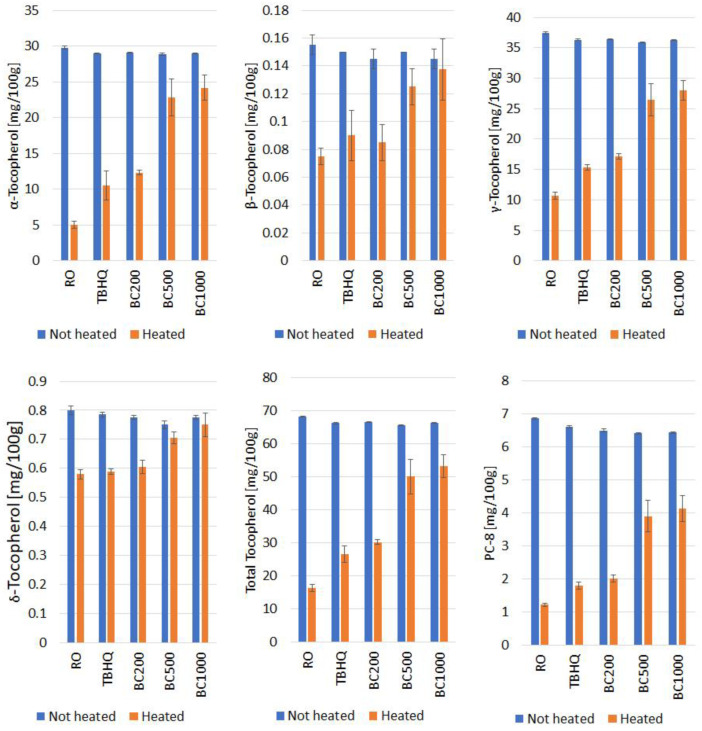
Changes of tocopherols content (mg/100 g) in not heated and heated rapeseed oil with the addition of black cumin essential oil. RO—rapeseed oil without addition, TBHQ—rapeseed oil with addition 200 ppm of TBHQ, BC200—rapeseed oil with addition 200 ppm of black cumin essential oil, BC500—rapeseed oil with addition 500 ppm of black cumin essential oil, BC1000—rapeseed oil with addition 1000 ppm of black cumin essential oil; 170RO, 170TBHQ, 170BC200, 170BC500, 170BC1000—rapeseed oils with addition of TBHQ and black cumin essential oil after 6 h of heating at 170 °C.

In the rapeseed oil used in the study, γ-tocopherol (37.42 mg/100 g) and α-tocopherol (29.81 mg/100 g) were present in the highest amounts, together constituting more than 98% of all tocopherols in the oil. PC-8 was also found in relatively high amounts (6.86 mg/100 g). The content of β- and δ-tocopherol was 0.16 and 0.80 mg/100 g of oil, respectively. The prepared mixtures of rapeseed oil with TBHQ and black cumin essential oil show similar contents of individual tocopherols (Figure 3). Heating the oils led to the degradation of tocopherols and to a reduction in their content. However, this phenomenon depended on the amount of additive used and the type of tocopherol. The greater degradation was observed for α- and δ-tocopherol and PC-8 in oil heated without additives. After 6 h heating at 170 °C, losses of 83.16%, 82.28%, and 71.48% were observed for α-tocopherol, PC-8, and δ-tocopherol, respectively. The loss of β-tocopherol was 51.61% and γ-tocopherol 27.50%. The addition of the synthetic antioxidant TBHQ and black cumin essential oil inhibited tocopherol degradation to varying degrees. However, the addition of the essential oil was more effective than the addition of TBHQ. The order of degradation inhibition was as follows: BC1000 > BC500 > BC200 > TBHQ > RO.

The addition of TBHQ and 200 ppm black cumin essential oil performed at similar levels. In the TBHQ-added oil, similar losses were observed for β-tocopherol (40%) and slightly higher for the other compounds. These were 65.4%, 57.84%, 25.16%, and 72.74% for α-, γ-, δ-, and PC-8, respectively. In the oil with 200 ppm of oil from black cumin, losses were 57.69%, 52.87%, 21.94% and 68.93% for α-, γ-, δ-, and PC-8, respectively. Additions of 500 and 1000 ppm of black cumin essential oil were the most effective, with total tocopherol losses of 25.96% and 19.84%, respectively. A significant reduction in the loss of all tocopherols and PC-8 was observed. The best effects were observed in the oil with the 1000 ppm addition, where the lowest losses were observed for δ- and β-tocopherol, 3.23% and 5.17%, respectively. Losses of other tocopherols and PC-8 were 16.64% for α-tocopherol, 22.83% for γ-tocopherol, and 35.68% for PC-8.

Thermal degradation of tocopherols is a common phenomenon in oils. Its rate depends on the type of oil (degree of unsaturation), the tocopherols composition, and the presence of other substances with antioxidant properties, as well as the process temperature and oxygen access [8,44]. The faster degradation of α-tocopherol compared to δ-tocopherol may be due to the much lower activation energy for the α-tocopherol molecule [45]. Thymoquinone present in the oil from black cumin may also be an important factor affecting the stability of tocopherols and the oxidative stability of the oil. In the study, the stability of oil from black cumin, pure triacylglycerols and tocopherols, and mixtures of tocopherols and thymoquinone were assessed [46]. Accelerated oxidative stability tests showed that the addition of thymoquinone to the triacylglycerols increased the induction time compared to the sample with tocopherols only. However, the sample with thymoquinone was significantly less stable than that with the synthetic antioxidant BHT. It should be noted that temperatures lower than 170 °C were used during the test, which may have improved the effectiveness of the synthetic antioxidants. The process temperature is also an important factor influencing the efficiency of black cumin essential oil and the degradation of tocopherols. A temperature of 200 °C essentially led to the complete degradation of tocopherols in the tested rapeseed oil samples with 100 and 200 ppm of black cumin essential oil. However, at 170 °C, despite the change in heating method (heating in a thin layer), similar properties of the essential oil and tocopherol behavior were observed [3].

### 3.6. Phytosterols Content

The phytosterol content of rapeseed oil was 7.18 mg/g oil. The main phytosterol was β-sitosterol (3.56 mg/g oil), followed by campesterol (2.62 mg/g oil). Brassicasterol (0.86 mg/g), campestanol (0.05 mg/g), and avenasterol (0.09 mg/g) were also identified in unheated oils. Heating the oils at 170 °C led to a slight reduction in the content of these compounds in the samples analyzed. The most significant changes were observed in β-sitosterol, campesterol, and brassicasterol. Changes in campestanol and avenasterol content were not statistically significant (Table 3). The total phytosterol content of the heated oils ranged from 6.65 mg/g to 6.99 mg/g oil. When analyzing the total phytosterol content of the oils after heating, no significant statistical differences were observed. The exception was the oil with the addition of 1000 ppm of black cumin essential oil. The final content of total phytosterols in this oil after heating was 6.99 mg/g oil. In rapeseed oil heated without additives, the highest decrease was observed for brassicasterol (10.47%), followed by campesterol (8.8%) and β-sitosterol (6.18%). A similar trend was observed in oils with TBHQ and 200 ppm black cumin essential oil. The losses were 13.5% and 15.7% for brassicasterol, 6.08% and 7.23% for campesterol, and 3.74% and 3.69% for β-sitosterol, respectively. For oils with the addition of 500 and 1000 ppm of black cumin essential oil, brassicasterol also exhibited the highest loss, followed by β-sitosterol and campesterol.

The loss of phytosterols during heating is linked to oxidation and polymerization processes, which can occur at different rates depending on the level of unsaturated fatty acid, process temperature, and oil surface-to-volume ratio [47,48]. The protective effect of natural antioxidants such as tocopherols should also be considered. Their presence in fatty systems subjected to heating led to inhibition of phytosterol oxidation [49,50]. Lower losses of phytosterols can therefore be linked to lower losses of tocopherols, particularly in the oils with 500 and 1000 ppm of black cumin essential oil.

### 3.7. Total Polar Compounds (TPC) and Polymerized Triacylglycerols (PTAG) Content

**Figure 4:**
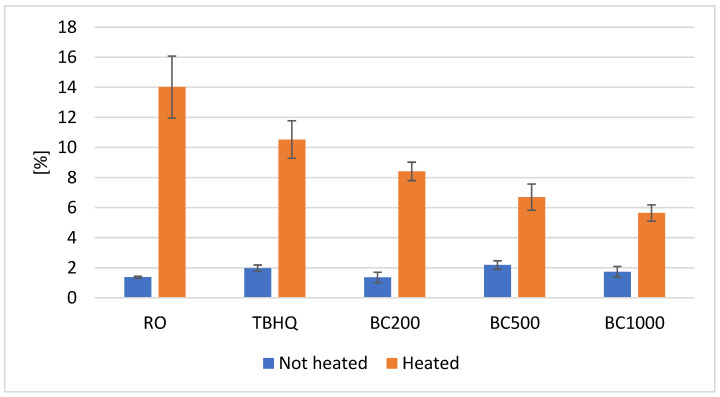
Changes of total polar content (%) in non-heated and heated rapeseed oil with the addition of black cumin essential oil. RO—rapeseed oil without addition, TBHQ—rapeseed oil with addition 200 ppm of TBHQ, BC200—rapeseed oil with addition 200 ppm of black cumin essential oil, BC500—rapeseed oil with addition 500 ppm of black cumin essential oil, BC1000—rapeseed oil with addition 1000 ppm of black cumin essential oil; 170RO, 170TBHQ, 170BC200, 170BC500, 170BC1000—rapeseed oils with addition of TBHQ and black cumin essential oil after 6 h of heating at 170 °C.

Under the influence of oxidation, hydrolysis, and thermal processes, numerous high- and low-molecular-weight compounds are formed in the oil during heating, most of which are polar in nature. An increase in their content leads to an increase in the polar fraction of oils. The total polar compound (TPC) content reflects the overall transformation of fats under high temperatures and is commonly used as an indicator of oil quality. In many countries, regulations have been implemented specifying maximum allowable levels of polar compounds in frying oils. Oils with a polar fraction content between 24% and 27% should be replaced. The total polar compounds content of non-heated and heated oils is shown in Figure 4.

The content of the polar fraction in the unheated oils ranged from 1.35 to 2.18%. Heating the prepared oils resulted in an increase in TPC content. In heated oils, TPC levels ranged from 5.64 to 14.02%. The highest TPC level was observed in the oil without antioxidant additives. The lowest level was characteristic of oil with the addition of 1000 ppm black cumin essential oil. The TPC content decreased with increasing amount of black cumin essential oil, and all oils with black cumin essential oil exhibited a lower polar fraction content than the oil with TBHQ. However, when considering the increase in polar fraction, calculated as the difference between the initial and final TPC contents, the samples can be ordered as follows: RO > BC200 > TBHQ > BC1000 > BC500. The increase in the polar fraction was 10.24 times, 5.35 times, 6.21 times, 3.07 times, and 3.26 times for the oil without any additive and the oils with TBHQ and 200, 500, and 1000 ppm of black cumin essential oil, respectively.

Triacylglycerol polymers (PTAG) are compounds with a higher molecular weight than a single triacylglycerol molecule. These polymers can be formed by a free-radical and non-radical mechanism. The free-radical mechanism is typical when fat is exposed to air during heating, e.g., during heating in a thin layer in a pan. In contrast, the non-radical mechanism can play a major role during the deep-frying process, where evaporating water can limit the access of oxygen to the fat [6]. The content of triacylglycerol dimers in the polar fraction of heated oils ranged from 3.48 to 26.43 mg/g oil. The highest content of triacylglycerol dimers was observed in the oil without antioxidant additives, while the lowest was found in the oil with 1000 ppm of black cumin essential oil (Table 4). The addition of TBHQ and 200 ppm of black cumin essential oil led to similar reductions in triacylglycerol dimers content (by 1.7–1.9 times). The results were not statistically different. Significantly stronger effects were obtained with higher additions of essential oil. With an addition of 500 ppm, the dimers content was reduced by 6.3 times, while at 1000 ppm, the reduction reached 7.6 times.

The reduction in the polar fraction, the slower increase in triacylglycerol polymers, and the slower degradation of fatty acids and tocopherols suggest that black cumin essential oil can be used as an additive to inhibit oil degradation during heating. The activity of black cumin oil was at a high level despite the fact that compounds in essential oils are often highly volatile, especially at elevated temperatures. However, most studies focus on pure essential oils, and the rate of transformation depends on the essential oil composition and heating conditions [25,51]. Higher stability of essential oils and the substances they contain, including terpenes, is observed when plant oils containing essential oils (natively or added) are heated [27]. This may be due to the protective role of the fat matrix, which reduces their volatility by binding them in the oil mass. Another factor limiting essential oils’ loss during heating may be the way the frying process is carried out. A major influence on the degradation of oils is the ratio of the volume of oil to the surface of contact with oxygen. This ratio is lower during pan frying (small volume, large surface area) and higher during deep frying (large volume, small surface area). Increasing this ratio and decreasing the surface area exposed to oxygen likely hinders the evaporation of essential oils by increasing their activity in deep-frying processes. However, only limited studies have addressed the thermal behavior of essential oils in vegetable oils and their limited loss in the fat matrix. Understanding the mechanisms occurring in fats during heating requires further research.

### 3.8. Principal Components Analysis (PCA)

**Figure 5:**
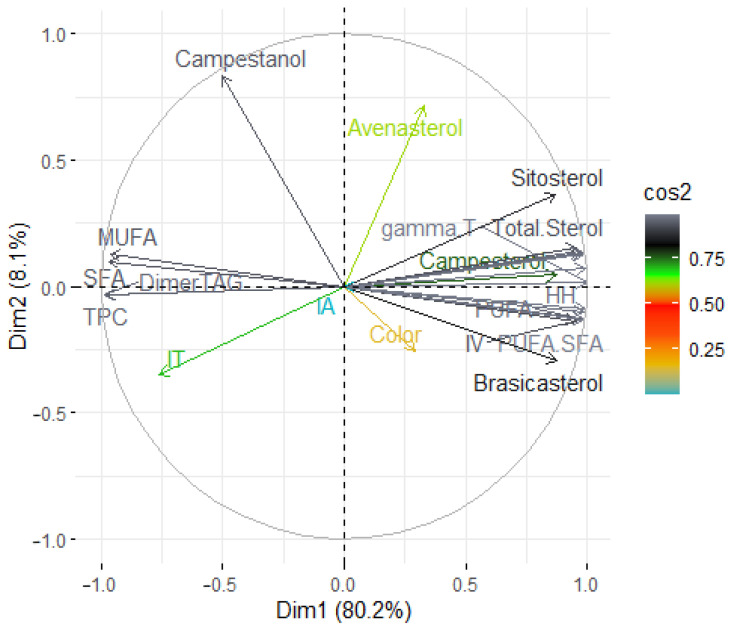

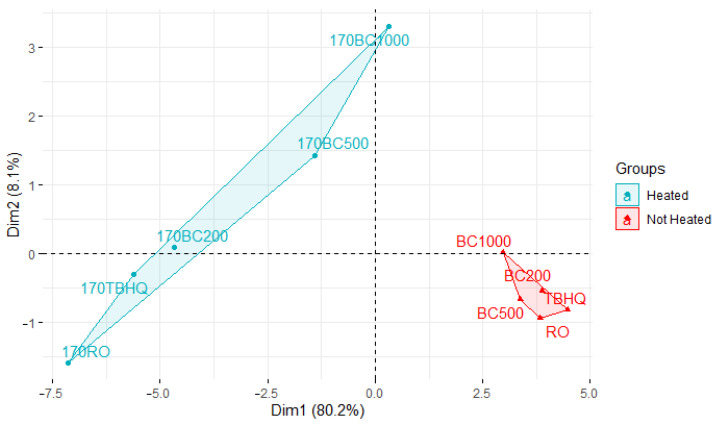
Principal component analysis (PCA) of the loadings plot and the score plot of data from fatty acid composition, indexes of lipid nutritional quality, tocopherols and phytosterol content, total polar compounds content and dimers of triacylglycerols in not heated and heated at 170 °C rapeseed oil without and with addition of 200 ppm of TBHQ and 200 ppm, 500 ppm and 1000 ppm of black cumin essential oil.

To investigate similarities and differences between the samples, Principal Component Analysis (PCA) was applied. The analysis covered both unheated and heated (at 170 °C) rapeseed oils, including those with TBHQ and varying levels of black cumin essential oil (200, 500, and 1000 ppm). The first two main factors comprised 88.3% (Dim1 = 80.2% and Dim2 = 8.1%) of the total variation. The results obtained by PCA show differences between non-heated and heated oil samples (Figure 5).

Based on the projection of the parameters into the loading plot, the first component was found to characterize most of the variability. The first factor (Dim 1) was positively correlated with share of PUFA (0.974), total tocopherols (0.992), total sterols (0.953), and negatively correlated with share of SFA (−0.966), MUFA (−0.963), content of polar compounds (−0.977), and triacylglycerol dimers (−0.984). The second factor (Dim 2) was mainly positively correlated with the content of campestanol (0.831). Based on the data presented in the score plot, a substantial variation between unheated and heated oils can be observed. In addition, considerable differentiation within the group of heated oils is observed. Two clusters are observed on the score plot. In the lower right quadrant, all unheated samples are located in close proximity to each other. This indicates their low variability. Oil samples heated at 170 °C are located on the left side of the graph. The samples without added antioxidants and with the addition of TBHQ are located under the *Y*-axis, and the samples with added black cumin essential oil are located above the *Y*-axis. The samples with the addition of TBHQ and 200 ppm of black cumin essential oil are at the smallest distance from each other, suggesting similar degradation profiles. These are samples characterized by similar fatty acid profiles and content of tocopherols and triacylglycerol dimers after heating. At a high distance from this group are the samples of oil with 500 ppm of black cumin essential oil. The extreme outlier is the sample with 1000 ppm addition, indicating the strongest protective effect during heating. These samples were characterized by lower levels of polar compounds and TAG dimers, and higher levels of natural antioxidants.

The PCA results indicate that the addition of black cumin essential oil significantly increases the oxidative stability of rapeseed oil during heating. In the multidimensional PCA space, oil samples with the addition of black cumin essential oils, especially those at 500 ppm and 1000 ppm, shift away from the cluster of unprotected, heated control samples and toward the region occupied by non-heated oils. This displacement reflects a pronounced reduction in degradation markers, namely total polar compounds and triacylglycerol dimers, coupled with the retention of bioactive constituents such as polyunsaturated fatty acids (PUFA), tocopherols, and sterols. Oil samples supplemented with black cumin essential oil therefore form a distinct cluster, characterized by a strong positive correlation with tocopherols and PUFA, and a strong negative correlation with polar compounds and dimers. These findings suggest that the addition of essential oil not only inhibits thermal degradation but also preserves the native oil components during heating. The stabilizing effect is thus evident not only in individual quality parameters but in their combined, multivariate profile. Such PCA-confirmed behavior can translate into important industrial advantages, including extended frying lifetimes due to slower degradation, reduced oil replacement frequency in commercial kitchens with associated cost savings, and the opportunity to market products with a clean-label claim owing to the natural origin of black cumin essential oils.

## 4. Conclusions

The use of black cumin essential oil in a stability study of heated rapeseed oil contributed to a reduction in undesired transformations. Compared to the synthetic antioxidant TBHQ, even the lowest addition of essential oil demonstrated comparable or slightly higher protective properties. Higher additions of black cumin essential oil stabilized rapeseed oil significantly more effectively than TBHQ. Oils enriched with essential oils showed lower loss of tocopherols and phytosterols, as well as smaller changes in fatty acid profile. Heated oils were also characterized by a lower increase in the polar fraction and the triacylglycerol dimers. An additional advantage of the essential oil used was its good solubility in oil. Regardless of the concentration used, the process was fast, and the oils obtained were homogeneous, without any precipitates or suspension. The use of essential oils in frying oils may be a promising strategy for improving the stability of frying oils. The addition of essential oils, including black cumin essential oil, may increase the price of the oils. However, the authors believe that the beneficial effects of inhibiting oil degradation during frying and the possibility of replacing synthetic antioxidants with natural compounds create a valuable opportunity for its use in oils for the food service industry. The use of black cumin essential oil in the formulation of frying oils may translate into significant industrial benefits, including extended frying lifetimes due to slower degradation, cost savings resulting from reduced frequency of oil replacement in commercial kitchens, and the opportunity to promote products with a clean-label claim due to the natural origin of black cumin essential oil.

In order to better evaluate such additives, further research is required to evaluate their behavior during prolonged heating or repeated frying of food products.

## Figures and Tables

**Table 1:** Chemical composition of black cumin essential oil.

No.	Compounds	Concentration (%)	Retention Time (min)	RI
1	α-thujene	15.92	9.575	934
2	α-pinene	5.83	9.823	942
3	β-pinene	5.90	11.196	988
4	α-phellandrene	0.10	12.044	1016
5	terpinolen	1.40	12.375	1027
6	p-cymene	32.84	12.594	1034
7	limonene	5.13	12.765	1040
8	cis-4-methoxythujane	2.38	14.810	1108
9	trans-4-methoxythujane	9.78	15.508	1132
10	terpinen-4-ol	1.24	17.371	1199
11	β-cyclocitral	1.08	17.897	1221
12	thymoquinone	14.49	19.075	1269
13	carvacrol	2.49	20.105	1321
14	α-longipinene	1.28	21.228	1383
15	longifolene	0.13	21.693	1413

**Table 2:** Changes of peroxide value, anisidine value, and acid value of fresh rapeseed oils without and with the addition of antioxidants.

	Peroxide Value (meq0_2_/kg Oil)	Anisidine Value	Acid Value (mg KOH/g Oil)
RO	1.03 ± 0.01 ^a^	0.01 ± 0.0 ^a^	0.06 ± 0.01 ^a^
TBHQ	1.00 ± 0.00 ^a^	0.01 ± 0.0 ^a^	0.06 ± 0.0 ^a^
BC200	1.06 ± 0.03 ^a^	0.01 ± 0.0 ^a^	0.06 ± 0.0 ^a^
BC500	1.09 ± 0.03 ^a^	0.01 ± 0.0 ^a^	0.06 ± 0.0 ^a^
BC1000	1.27 ± 0.06 ^b^	0.01 ± 0.0 ^a^	0.06 ± 0.0 ^a^

Data are expressed as means ± SD from two independent determinations. Different lowercase letters in the same row indicate statistically significant differences at *p* < 0.05. Values are means of two determinations ± SD. RO—rapeseed oil without addition, TBHQ—rapeseed oil with addition 200 ppm of TBHQ, BC200—rapeseed oil with addition 200 ppm of black cumin essential oil, BC500—rapeseed oil with addition 500 ppm of black cumin essential oil, BC1000—rapeseed oil with addition 1000 ppm of black cumin essential oil.

**Table 3:** Changes in sterols content (mg/g oil) in non-heated and heated rapeseed oil with the addition of black cumin essential oil.

Sterols	RO		TBHQ		BC200		BC500		BC1000	
	Not Heated	Heated	Not Heated	Heated	Not Heated	Heated	Not Heated	Heated	Not Heated	Heated
Brassicasterol	0.86 ± 0.03 ^a^	0.77 ± 0.02 ^bc^	0.89 ± 0.01 ^a^	0.77 ± 0.02 ^bc^	0.89 ± 0.03 ^a^	0.75 ± 0.03 ^b^	0.87 ± 0.00 ^a^	0.76 ± 0.02 ^b^	0.82 ± 0.02 ^abc^	0.81 ± 0.02 ^ac^
Campesterol	2.62 ± 0.01 ^ab^	2.39 ± 0.04 ^c^	2.63 ± 0.01 ^ab^	2.47 ± 0.06 ^abc^	2.65 ± 0.02 ^b^	2.44 ± 0.09 ^ac^	2.52 ± 0.02 ^abc^	2.49 ± 0.07 ^abc^	2.51 ± 0.00 ^abc^	2.49 ± 0.06 ^ab^
Campestanol	0.05 ± 0.00 ^a^	0.06 ± 0.01 ^a^	0.05 ± 0.01 ^a^	0.06 ± 0.01 ^ab^	0.05 ± 0.00 ^ab^	0.07 ± 0.00 ^ab^	0.05 ± 0.00 ^ab^	0.07 ± 0.01 ^ab^	0.06 ± 0.00 ^ab^	0.08 ± 0.01 ^b^
β-sitosterol	3.56 ± 0.05 ^ab^	3.34 ± 0.04 ^a^	3.48 ± 0.06 ^ab^	3.35 ± 0.05 ^a^	3.52 ± 0.01 ^ab^	3.39 ± 0.14 ^ab^	3.55 ± 0.03 ^ab^	3.46 ± 0.12 ^ab^	3.55 ± 0.01 ^ab^	3.58 ± 0.09 ^b^
Avenasterol	0.09 ± 0.00 ^a^	0.09 ± 0.00 ^a^	0.10 ± 0.00 ^a^	0.10 ± 0.01 ^a^	0.10 ± 0.01 ^a^	0.09 ± 0.01 ^a^	0.10 ± 0.01 ^a^	0.1 ± 0.00 ^a^	0.10 ± 0.00 ^a^	0.11 ± 0.01 ^a^
Total	7.18 ± 0.03 ^ac^	6.65 ± 0.06 ^b^	7.16 ± 0.08 ^abc^	6.75 ± 0.10 ^ab^	7.21 ± 0.05 ^ac^	6.74 ± 0.27 ^ab^	7.09 ± 0.00 ^abc^	6.87 ± 0.17 ^abc^	7.04 ± 0.03 ^abc^	6.99 ± 0.09 ^c^

Data are expressed as means ± SD from four independent determinations. Different lowercase letters in the same row indicate statistically significant differences at *p* < 0.05.RO—rapeseed oil without addition. TBHQ—rapeseed oil with the addition of 200 ppm of TBHQ. BC200—rapeseed oil with the addition of 200 ppm of black cumin essential oil. BC500—rapeseed oil with the addition of 500 ppm of black cumin essential oil. BC1000—rapeseed oil with the addition of 1000 ppm of black cumin essential oil.

**Table 4:** Changes of triacylglycerols dimers content [mg/g oil] in non-heated and heated rapeseed oil with the addition of black cumin essential oil.

	RO	TBHQ	BC200	BC500	BC1000
Not heated	-	-	-	-	-
Heated	26.43 ± 1.33 ^a^	15.24 ± 0.78 ^b^	13.77 ± 0.72 ^b^	4.20 ± 0.28 ^c^	3.48 ± 0.36 ^d^

Data are expressed as means ± SD from four independent determinations. Different lowercase letters in the same row indicate statistically significant differences at *p* < 0.05. RO—rapeseed oil without addition. TBHQ—rapeseed oil with the addition of 200 ppm of TBHQ. BC200—rapeseed oil with the addition of 200 ppm of black cumin essential oil. BC500—rapeseed oil with the addition of 500 ppm of black cumin essential oil. BC1000—rapeseed oil with the addition of 1000 ppm of black cumin essential oil.

## Data Availability

The original contributions presented in the study are included in the article, further inquiries can be directed to the corresponding authors.
